# Intranasal Administration of KYCCSRK Peptide Rescues Brain Insulin Signaling Activation and Reduces Alzheimer’s Disease-like Neuropathology in a Mouse Model for Down Syndrome

**DOI:** 10.3390/antiox12010111

**Published:** 2023-01-02

**Authors:** Antonella Tramutola, Simona Lanzillotta, Giuseppe Aceto, Sara Pagnotta, Gabriele Ruffolo, Pierangelo Cifelli, Federico Marini, Cristian Ripoli, Eleonora Palma, Claudio Grassi, Fabio Di Domenico, Marzia Perluigi, Eugenio Barone

**Affiliations:** 1 Department of Biochemical Sciences “A. Rossi-Fanelli”, Sapienza University of Rome, Piazzale A. Moro 5, 00185 Roma, Italy; 2 Department of Neuroscience, Università Cattolica del Sacro Cuore, 00168 Roma, Italy; 3 Fondazione Policlinico Universitario A. Gemelli, Istituto di Ricovero e Cura a Carattere Scientifico, 00168 Roma, Italy; 4 Department of Physiology and Pharmacology, Istituto Pasteur-Fondazione Cenci Bolognetti, University of Rome Sapienza, 00185 Rome, Italy; 5 IRCCS San Raffaele Roma, 00163 Rome, Italy; 6 Department of Applied Clinical and Biotechnological Sciences, University of L’Aquila, 67100 L’Aquila, Italy; 7 Department of Chemistry, Sapienza University of Rome, Piazzale A. Moro 5, 00185 Roma, Italy

**Keywords:** Alzheimer’s disease, brain insulin resistance, Down syndrome, DYRK1A, intellectual disability

## Abstract

Down syndrome (DS) is the most frequent genetic cause of intellectual disability and is strongly associated with Alzheimer’s disease (AD). Brain insulin resistance greatly contributes to AD development in the general population and previous studies from our group showed an early accumulation of insulin resistance markers in DS brain, already in childhood, and even before AD onset. Here we tested the effects promoted in Ts2Cje mice by the intranasal administration of the KYCCSRK peptide known to foster insulin signaling activation by directly interacting and activating the insulin receptor (IR) and the AKT protein. Therefore, the KYCCSRK peptide might represent a promising molecule to overcome insulin resistance. Our results show that KYCCSRK rescued insulin signaling activation, increased mitochondrial complexes levels (OXPHOS) and reduced oxidative stress levels in the brain of Ts2Cje mice. Moreover, we uncovered novel characteristics of the KYCCSRK peptide, including its efficacy in reducing DYRK1A (triplicated in DS) and BACE1 protein levels, which resulted in reduced AD-like neuropathology in Ts2Cje mice. Finally, the peptide elicited neuroprotective effects by ameliorating synaptic plasticity mechanisms that are altered in DS due to the imbalance between inhibitory vs. excitatory currents. Overall, our results represent a step forward in searching for new molecules useful to reduce intellectual disability and counteract AD development in DS.

## 1. Introduction

Down syndrome (DS) is the most frequent genetic cause of intellectual disability and is strongly associated with Alzheimer’s disease (AD) [1]. It is a multifaceted disorder with over 80 clinically defined phenotypes including those affecting the central nervous system, heart, gastrointestinal tract, skeleton, and immune system [2]. Phenotypes associated with trisomy 21 vary in both incidence and severity, leading to a vast array of phenotypic combinations [3]. Prevalence of overweight can reach 70% in subjects with DS leading to a higher incidence of type 2 diabetes mellitus (T2DM) and/or obesity [4]. Metabolic disorders are characterized by alterations in cell metabolism, e.g., mitochondrial defects, increased oxidative stress levels, impaired glucose, and lipid metabolism, finally resulting in reduced energy production and cellular dysfunctions that account for a higher incidence of diabetes [5,6] and/or obesity [7].

An intriguing epidemiological correlation between obesity, glucose dysmetabolism and diabetes, and various complex brain diseases, including AD, exists. Diabetic subjects develop insulin resistance, which leads to an increased risk of developing AD in later life [8,9,10]. In a perfect storm, neuroinflammation, oxidative stress, and mitochondrial dysfunction would aggravate brain insulin resistance and amyloid-beta (Aβ) accumulation in brain lesions [8,10,11,12].

Remarkably, recent studies highlight that brain insulin resistance can develop even independently from peripheral alterations both during aging and in AD, though the molecular mechanisms are similar. Decreased insulin concentrations and insulin receptor binding were reported in the cortex of elderly individuals without dementia [10,13] and in AD patients without T2DM [14,15,16,17,18]. In two independent cohorts of post-mortem brain samples isolated from individuals with AD or mild cognitive impairment (MCI), substantial abnormalities were described in the basal activation of insulin signaling [14]. These abnormalities were negatively correlated with global cognition and memory scores regardless of Aβ and Tau levels [16], suggesting that brain insulin resistance contributes to cognitive impairment independently from AD neuropathology [15]. In support of these observations, the evaluation of neuronal-derived extracellular vesicles (nEVs) cargo demonstrated that increased markers of brain insulin resistance in nEVs predict the development of AD in elderly individuals [19].

DS recapitulates several risk factors associated with brain insulin resistance development, i.e., increased Aβ production and deposition due to triplication of the amyloid precursor protein gene *APP* mapping to human chromosome 21 (HSA21), and increased rates of obesity, glucose intolerance, and T2DM. Intriguingly, our group showed the accumulation of insulin resistance markers in the brain in DS individuals already in childhood, regardless of metabolic disorders, and even before AD development [4,20,21,22,23]. Furthermore, brain insulin resistance manifests along with an impairment of cell energy metabolism before frank AD pathological hallmarks accumulation both in humans and DS mouse models [22,23], suggesting that a close link exists among these alterations that contribute to brain dysfunctions and likely favor the development of AD in DS.

Complex disorders pose tremendous challenges to research and healthcare. The increasing number of patients with co- and multi-morbidities causes an urgent need to improve care but also to develop new strategies to assess the progression of diseases, and define socio-economic and lifestyle contributing factors. As brain insulin resistance has been identified as a risk factor for AD, the concept has developed that drugs used to treat peripheral insulin resistance (i.e., in T2DM or obesity) may have neuroprotective properties [24,25]. Among the strategies to ameliorate the activation of insulin signaling in the brain, intranasal administration of antidiabetic drugs is under evaluation in the field of AD [26,27,28,29]. Intranasal administration represents an effective strategy that allows drugs to bypass the blood-brain barrier (BBB) and directly reach the brain, thereby avoiding side effects caused by systemic administration [30]. To note, intranasal insulin administration promoted neuroprotective effects resulting in improved cognitive functions in MCI and AD subjects [31,32,33,34,35]. Moreover, a recent pilot study showed that a single dose of intranasal insulin was safe in DS individuals and promoted a trend toward improved performance on memory retention [36], although further investigations are required due to the very small sample size of the cohort of patients.

Successful intranasal delivery of biologics such as peptides, proteins, monoclonal antibodies, oligonucleotides, and gene and cell therapies via the nose-to-brain route are of increasing interest due to their high potency and selectivity [37,38]. Peptides biodegrade into non-toxic metabolites, possess a minimal potential for drug-drug interactions, are less likely to cause an immunogenic reaction when compared to larger proteins, and have lower production costs [37,38,39]. These favorable properties have resulted in peptides having a good probability of securing regulatory approval when compared to low molecular weight drugs [38]. Moreover, their small size enables peptides to penetrate the cell membrane to target intracellular molecules [39].

In searching for new potential neuroprotective molecules for DS, we focused on the effects promoted by the KYCCSRK peptide. Previous works described an unprecedented approach for stimulating insulin signaling activation and glucose uptake by means of the KYCCSRK peptide, corresponding to the C-terminal 7 residues (K^291^YCCRSK) of the human biliverdin reductase-A (BVR-A) protein [27,40,41], this latter being a key player in the regulation of insulin signaling [42,43]. The KYCCSRK peptide was shown to promote both the activation of the insulin receptor (IR) kinase activity and the activation of ERK1/2 and AKT downstream from IR [40,41] on its own in HEK cells, thus representing a promising approach to counteract insulin resistance. Intriguingly, the KYCCSRK acts predominantly at the intracellular level by favoring conformational changes of the IR kinase domain in the β subunits and/or by interacting with ERK1/2 and AKT, thus triggering their activation in the absence of insulin [40,41]. However, none of the above-mentioned studies addressed the effects of the KYCCSRK peptide in vivo, neither in the brain.

In the current work, we tested the hypothesis that the intranasal administration of KYCCSRK peptide rescues brain insulin signaling activation and promotes neuroprotective effects in the brain of Ts2Cje mice (a model for DS).

## 2. Materials and Methods

### 2.1. Mouse Colony

Ts2Cje (Rb(12.Ts171665Dn)2Cje) mice are a well-established murine model of DS characterized by a triple copy of a Robertsonian fusion chromosome carrying the distal end of Chr16 and Chr12. Parental generations were purchased from Jackson Laboratories (Bar Harbour, ME, USA). The mouse colony was raised by a crossbreed of Ts2Cje trisomic females with euploid (B6EiC3SnF1/J) F1 hybrid males (Eu). The parental generations were purchased from Jackson Laboratories (Bar Harbour, ME, USA). These breeding pairs produce litters containing both trisomic (Ts2Cje) and euploid (Eu) offspring. Pups were genotyped to determine trisomy by standard PCR, using Reinoldth’s method [44]. Mice were housed in clear Plexiglas cages (20 × 22 × 20 cm) under standard laboratory conditions with a temperature of 22 ± 2 °C and 70% humidity, a 12-h light/dark cycle, and free access to food and water.

### 2.2. KYCCSRK Treatment

Nine-month old male Ts2Cje and Eu mice were used in this study based on previous results showing increased accumulation of brain insulin resistance and AD neuropathological hallmarks at this age [22]. Euploid (Eu) and Ts2Cje (Ts-V) mice were treated with vehicle (saline) (5 μL/nostril), and an additional group of Ts2Cje (Ts-P) mice received an intranasal administration of 0.5 mM N-myristoylated KYCCSRK peptide (hereafter KYCCSRK) [PO#SP161119 Myr-KYCCSRK, (Biomatik, Wilmington, DE, USA)] for two weeks (n = 4/group). The choice to use a myristoylated peptide relies on the concept that myristoylated peptides cross the plasma membrane and can modulate intracellular processes, i.e., insulin signaling, as previously reported [40,41]. Based on the results collected in a pilot dose-response treatment (0, 0.12, 0.25, 0.5, 1, and 2 mM) in Eu mice (n = 3/group), we selected the dose of 0.5 mM, which was effective in stimulating the insulin signaling in the frontal cortex, as demonstrated by the significantly increased activation of the AKT-AS160-GLUT4 pathway (see Supplementary Figure S1).

### 2.3. Primary Neurons

For primary neuronal culture, Ts2cje and euploid pups at 0–1 post-natal day were used. Regarding the collection of Ts2Cje primary neurons, because the breeding pairs produce litters containing both trisomic (Ts2Cje) and euploid (Eu) offsprings, Ts2Cje pups were selected by tail-genotyping. After the sacrifice, the cortex was dissected and processed (Ts2Cje and Eu separately) following a specific procedure to obtain neuronal cells. In detail, cortical tissues were mechanically dissociated in cold phosphate-buffered saline (PBS) supplemented with Ca^2+^ and Mg^2+^ (Sigma-Aldrich, St. Louis, MO, USA) and centrifuged at 1100 rpm for 3 min at room temperature. The supernatant was removed and a solution of PBS w/o Ca^2+^ and Mg^2+^ [Sigma-Aldrich, St. Louis, MO, USA] and 0.25% Trypsin (GIBCO, Thermo Fisher Scientific, Waltham, MA USA) was added for chemical tissue dissociation in a shaking water bath at 37 °C for 10 min. To inactivate the trypsin, 10% fetal bovine serum (FBS) (GIBCO, Thermo Fisher Scientific, Waltham, MA, USA) was added to the dissociated tissues and centrifuged at 1100 rpm for 3 min at room temperature. The cell-pellet was resuspended in Minimum Essential Medium (MEM) (GIBCO, Thermo Fisher Scientific, Waltham, MA USA) supplemented with 1% FBS, 1% L-glutamine (200 mM, Sigma-Aldrich, St. Louis, MO, USA), 1% glucose (25 mM, Sigma-Aldrich, St. Louis, MO, USA) and 1% Gentamicin (0,1 mg/mL, Sigma-Aldrich, St. Louis, MO, USA) and centrifuged at 1100 rpm for 10 min at room temperature. The obtained pellet was resuspended in a second MEM supplemented with 5% FBS, 5% human serum, 1% L-glutamine (200 mM), 1% glucose (25 mM), and 1% gentamycin (0.1 mg/mL), and the cells were plated at a density of 150,000 cells/well in a multi-well previously coated with poly-L-lysine (Sigma-Aldrich, St. Louis, MO, USA). After 24 h the medium was replaced with Neurobasal Medium (GIBCO, Thermo Fisher Scientific, Waltham, MA, USA) supplemented with 2% B-27 serum free (GIBCO, Thermo Fisher Scientific, Waltham, MA, USA), 1% L-glutamine (200 mM, Sigma-Aldrich, St. Louis, MO, USA), and 1% gentamicin (0.1 g/mL, Sigma-Aldrich, St. Louis, MO, USA) to start neuronal differentiation. After 4 days, the medium was replaced with Neurobasal Medium supplemented with 2% B-27 (GIBCO, Thermo Fisher Scientific, Waltham, MA, USA) and 1% gentamicin (0,1 mg/mL, Sigma-Aldrich, St. Louis, MO, USA) to let the neurons grow up. After 10 days from the cell plating, the neurons were treated with the KYCCSRK peptide at different doses (1, 5, and 20 μM) as previously described [27,40] (see Supplementary Figure S2). After 24 h cells were collected and stored at −80 °C for the Western blot analysis described below.

### 2.4. Samples Preparation

Total protein extracts from either cortical primary neurons or Ts2Cje and Eu cortical samples were prepared in radioimmunoprecipitation assay (RIPA) buffer (pH 7.4) containing 50 mM Tris-HCl (pH 7.4, Sigma-Aldrich, St. Louis, MO, USA), 150 mM NaCl (Sigma-Aldrich, St. Louis, MO, USA), 1% NP-40 (Sigma-Aldrich, St. Louis, MO, USA), 0.25% sodium deoxycholate (Sigma-Aldrich, St. Louis, MO, USA), 1 mM EDTA (Sigma-Aldrich, St. Louis, MO, USA), 0.1% sodium dodecyl sulfate (SDS, Sigma-Aldrich, St. Louis, MO, USA) and supplemented with phosphatase and protease inhibitors (1:100, Sigma-Aldrich, St. Louis, MO, USA). Then, samples were homogenized by 20 strokes of a Wheaton tissue homogenizer, sonicated, and centrifuged at 14,000 rpm for 30 min at 4 °C to remove debris. The supernatant was collected, and the total protein concentration was determined by the BCA method according to manufacturer’s instructions (Thermo Fisher Scientific, Waltham, MA, USA).

### 2.5. Western Blot

Fifteen μg of proteins were resolved via SDS-PAGE using Criterion™ TGX Stain-Free™ precast gel (Bio-Rad Laboratories, Hercules, CA, USA) in a Criterion large format electrophoresis cell (Bio-Rad Laboratories, Hercules, CA, USA) in Tris/Glycine/SDS (TGS) Running Buffer (Bio-Rad Laboratories, Hercules, CA, USA). Immediately after electrophoresis, the gel was placed on a Chemi/UV/Stain-Free tray and then placed into a ChemiDoc MP imaging System (Bio-Rad Laboratories, Hercules, CA, USA) and UV-activated based on the appropriate settings with Image Lab Software (Bio-Rad Laboratories, Hercules, CA, USA) to collect total protein load image. Following electrophoresis and gel imaging, the proteins were transferred onto a nitrocellulose membrane by Trans-Blot Turbo Transfer System (Bio-Rad Laboratories, Hercules, CA, USA). To prove the transfer, the blot was imaged by the ChemiDoc MP imaging system using the Stain-Free Blot settings. The nitrocellulose membrane was blocked using 3% BSA (SERVA Electrophoresis GmbH, Heidelberg, Germany) in 1X Tris Buffer Saline (TBS) containing 0.01% Tween20 (Sigma-Aldrich, St. Louis, MO, USA) and incubated overnight at 4 °C with the primary antibodies listed in Table 1. The day after, all membranes were washed with 1X TBS containing 0.01% Tween20 (Sigma-Aldrich, St. Louis, MO, USA) and incubated at room temperature for 1 h with the respective secondary antibody conjugated with horseradish peroxidase: anti-rabbit (1:10,000; Bio-Rad Laboratories, Hercules, CA, USA), anti-mouse (1:10,000; Bio-Rad Laboratories, Hercules, CA, USA). Membranes were developed with Clarity enhanced chemiluminescence (ECL) substrate (Bio-Rad Laboratories, Hercules, CA, USA) and then acquired with Chemi-Doc MP (Bio-Rad, Hercules, CA, USA) and analyzed using Image Lab 6.1 software (Bio-Rad, Hercules, CA, USA) that allows the normalization of a specific protein signal by the mean of total proteins load. Total protein staining measures the aggregate protein signal (sum) in each lane and eliminates the error that can be introduced by a single internal control protein. Total protein staining is a reliable and widely applicable strategy for quantitative immunoblotting. It directly monitors and compares the aggregate amount of sample protein in each lane, rather than using an internal reference protein as a surrogate marker of sample concentration. This direct, straightforward approach to protein quantification may increase the accuracy of normalization. Total load can be detected taking advantage of the Stain free technology (Bio-Rad, Hercules, CA, USA). Stain-free imaging technology utilizes a proprietary trihalo compound to enhance natural protein fluorescence by covalently binding to tryptophan residues with a brief UV activation. Images of the gel or membrane after transfer can easily be captured multiple times without staining and de-staining steps.

### 2.6. Slot Blot

For the analysis of total protein carbonyls (PC) levels, 5 μL of cortical total protein extract samples were derivatized with 5 μL of 10 mM 2,4-dinitrophenylhydrazine (DNPH, OxyBlot™ Protein Oxidation Detection Kit, Merck-Millipore, Darmstadt, Germany) in the presence of 5 μL of 10% sodium dodecyl sulfate (SDS) for 20 min at room temperature. The samples were then neutralized with 7.5 μL of neutralization solution (2 M Tris in 30% glycerol) and loaded onto a nitrocellulose membrane as described below.

For total (i) protein-bound 4-hydroxy-2-nonenals (HNE) and (ii) 3-nitrotyrosine (3-NT) levels: 5 μL of cortical total protein extract samples, 5 μL of 12% SDS, and 5 μL of modified Laemmli buffer containing 0.125 M Tris base (pH 6.8), 4% (*v*/*v*) SDS, and 20% (*v*/*v*) glycerol were incubated for 20 min at room temperature and then loaded onto nitrocellulose membrane as described below.

Proteins (250 ng) were loaded in each well on a nitrocellulose membrane under vacuum using a slot blot apparatus. Membranes were blocked for 1 h with a solution of 3% (*w*/*v*) BSA in PBS containing 0.01% (*w*/*v*) sodium azide and 0.2% (*v*/*v*) Tween 20 and incubated respectively with primary antibodies anti-HNE and anti-3NT (details are listed in Table 1) for 2 h at RT. Membranes were washed in TTBS following primary antibody incubation three times at intervals of 5 min each and then incubated with anti-rabbit or mouse IgG alkaline phosphatase secondary antibodies (Sigma-Aldrich, St Louis, MO, USA) for 1 h at room temperature. Then, membranes were washed three times in TBS solution containing 0.01% Tween 20 for 5 min each and developed with Sigma fast tablets (5-bromo-4-chloro-3-indolyl phosphate/nitroblue tetrazolium substrate [BCIP/NBT substrate]). Membranes were dried and the images were acquired using the ChemiDoc XP image system and analyzed using Image Lab software (Bio-Rad Laboratories, Hercules, CA, USA). No non-specific binding of the antibody to the membrane was observed.

### 2.7. Real-Time PCR

RNA was obtained from cortical tissue of Ts2Cje and Eu mice treated with vehicle or the KYCCSRK peptide. Tissues were lysed with an appropriate volume of QIAzol Reagent (QIAzol Lysis Reagent, Qiagen, Hilden, Germany). Subsequently, chloroform was added (1:5, Sigma-Aldrich, St. Louis, MO, USA), and samples were kept for 3 min at RT before being centrifuged at 12,000 rpm and 4 °C for 15 min. Following that, isopropanol (100%, Sigma-Aldrich, St. Louis, MO, USA) was added to each sample at RT for 10 min to separate RNA. Then, samples were centrifuged at 12,000 rpm and 4 °C for 15 min. The supernatant was discarded, and pellets were washed with 75% ethanol (Sigma-Aldrich, St. Louis, MO, USA) followed by further centrifugation at 7500 rpm and 4 °C for 5 min. The pellets were resuspended in RNAse-free H_2_O (Sigma-Aldrich, St. Louis, MO, USA). The RNA was quantified using the Biospec Nano reverse transcribed using the cDNA High-Capacity kit (Applied Biosystems, Foster City, CA, USA), including reverse transcriptase, random primers, and buffer according to the manufacturer’s instructions. The cDNA was produced through a series of heating and annealing cycles in the MultiGene OPTIMAX 96-well Thermocycler (LabNet International, Edison, NJ, USA). Real-time PCR was carried out using the SensiFAST™ SYBR^®^ and No-ROX Kit (Bioline, London, UK) in a CFX Connect Real-Time PCR machine (Bio-Rad Laboratories, Hercules, CA, USA). Primers used for the RT-PCR are DYRK1A FW: 5′-TGGGGCAGAGGATATACCAGT-3′ and RV: 5′- GTCGATAGCAAGGTCATAAGGCA-3′ and BACE1 FW 5′-GGATTATGGTGGCCTGAGCA-3′ RV 5′-CACGAGAGGAGACGACGATG-3′ (BACE1: NM_011792.7; DYRK!A: NM_007890.2).

### 2.8. Membrane Preparation, Microtransplantation, and Xenopus Oocytes Recordings

Xenopus oocytes were obtained from adult female frogs. The procedure for the oocytes’ preparation was described elsewhere [45]. Then, the cytoplasmatic injection was performed with a pressure microinjector (PLI-100, Warner Instruments, Holliston, MA, USA). Cell membranes were prepared from cortex of Eu, Ts-V, and Ts-P mice (n = 4/group) that were immediately processed after their removal or stored at −80 °C. Then, the injected oocytes were maintained in modified Barth’s solution at 16 °C until electrophysiological recordings were performed. Xenopus frogs were purchased from Xenopus1 (USA). The use of *Xenopus laevis* frogs, and the surgical procedures for oocytes preparation and use conformed to the Italian Ministry of Health guidelines and were approved by the same institution (authorization no 427/2020-PR).

### 2.9. Voltage-Clamp Recordings

Experiments with microtransplanted oocytes were carried out 24–48 h after cytoplasmic injection and GABA and AMPA-evoked currents (I_GABA_ and I_AMPA_) were recorded using the technique of two-electrode voltage clamp. The microelectrodes were filled with 3 M KCl [46] and oocytes placed in a recording chamber (0.1 mL volume) continuously perfused with oocyte Ringer solution (OR: 82.5 mM NaCl; 2.5 mM KCl; 2.5 mM CaCl2; 1 mM MgCl2; 5 mM Hepes, adjusted to pH 7.4 with NaOH) at room temperature (20–22 °C). The timing of neurotransmitter application was controlled by a gravity driven multi-valve perfusion system (9–10 mL/min) which was controlled through a computer interface (Biologique RSC- 200; Claix, France) to ensure the exact duration of each application. With our setup, 0.5 to 1 s was enough to completely replace the entire volume of the applied solution in the recording chamber.

In these experiments, 500 µM GABA was applied for 5 s followed by a washout of 4–5 min. To record AMPA currents, we used a preincubation of 20 s with cyclothiazide (CTZ, 20 μM to avoid AMPA’s receptor desensitization) before applying 20 μM AMPA plus CTZ for 10 s [47]. The stability of the I_GABA_ and I_AMPA_ was ascertained by performing two consecutive GABA and AMPA applications, separated by a 4–5 min washout. The cells that had a <5% variation of current amplitude were considered in the statistical analysis.

### 2.10. Statistical Analyses

Statistical analyses were performed by using one-way ANOVA analysis with Dunnett’s or Kruskal–Wallis multiple comparison tests for the evaluation of differences between more than two groups. All statistical tests were two-tailed and the level of significance was set at 0.05. Data are expressed as mean ± SEM per group. Correlation analyses were calculated by Pearson’s rank correlations and displayed as a correlation matrix. Furthermore, principal component analysis (PCA) was used. PCA is a statistical method for data compression and visualization. Multivariate data are projected along orthogonal directions (principal components) along which they possess the maximum variance. Similarity and dissimilarities among samples are inspected by plotting the new coordinates of the data along the principal components (scores), while interpretation of the observed differences is achieved by investigating the variable contributions to the new directions (loadings). More specifically, PCA analysis was conducted on the data matrix after column autoscaling by means of in-house written routines running under Matlab (R2015b; The Mathworks, Natick, MA, USA) environment. All the other statistical analyses were performed using Graph Pad Prism 9.0 software (GraphPad, La Jolla, CA, USA).

## 3. Results

### 3.1. Intranasal KYCCSRK Administration Rescues Insulin Signaling Activation in the Frontal Cortex of Ts2Cje Mice

**Figure 1:**
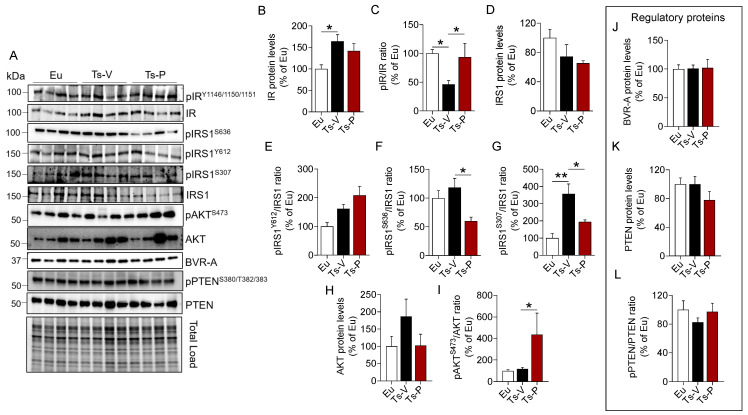
Activation of insulin signaling following KYCCSRK intranasal administration in Ts2Cje mice. IR, IRS1, AKT, BVR-A, and PTEN protein levels and phosphorylation were evaluated in the frontal cortex of Eu and Ts2Cje (Ts-V) mice treated with vehicle (saline) and Ts2Cje (Ts-P) treated with KYCCSRK peptide (0.5 mM) for two weeks (n = 4/group). (**A**) Representative Western blot and total load images and densitometric evaluation of (**B**,**C**) IR protein levels and activation [evaluated as pIR^Y1146/1150/1151^/IR ratio], (**D**) IRS1 protein levels, (**E**) IRS1 activation evaluated as pIRS1^Y612^/IRS1 ratio], (**F**,**G**) IRS1 inhibition [evaluated as pIRS1^S636^/IRS1 and pIRS1^S307^/IRS1 ratio], (**H**,**I**) AKT protein levels and activation (evaluated as pAKT^S473^/AKT ratio), (**J**) BVR-A protein levels, and (**K**,**L**) PTEN protein levels and inhibition (evaluated as pPTEN^S380/T382/383^/PTEN). All densitometric values were normalized per total protein load and are given as a percentage of Eu set as 100%. Data are shown as mean ± SEM. One-way ANOVA with Dunnett test: * *p* < 0.05, ** *p* < 0.01.

To evaluate the effects of the intranasal KYCCSRK peptide administration on the brain insulin signaling pathway in Ts2Cje mice, we started by measuring protein levels and activation of IR and IRS1, the latter being the main target of IR kinase activity [10]. IR protein levels were significantly increased in Ts-V compared to Eu mice (+64%, *p* = 0.021), while no changes were induced by KYCCSRK peptide treatment (Ts-P) (Figure 1B). Activation of IR (evaluated as pIRY1146/1150/1151/IR ratio) was significantly reduced in Ts-V with respect to Eu mice (−54% vs. Eu, *p* = 0.023), while the KYCCSRK administration rescued IR activation in Ts-P mice (+64%, *p* = 0.014) (Figure 1C). Regarding IRS1 total protein levels, no significant changes were observed among groups (Figure 1D). No changes for IRS1 activation (evaluated as pIRS1Y612/IRS1 ratio) were observed (Figure 1E). IRS1 inhibition was evaluated by the mean of S636 and S307 phosphorylation sites (reported as pIRS1S636/IRS1 and pIRS1S307/IRS1 ratio, respectively), which are well known markers of insulin resistance [48,49]. We found that S636 phosphorylation levels were not different between Ts-V and Eu, while S307 phosphorylation levels were significantly increased in Ts-V mice (+258% vs. Eu, *p* = 0.001) (Figure 1F,G). Rather, the intranasal KYCCSRK treatment led to a consistent reduction of both S636 (−60% vs. Ts-V, *p* = 0.01) and S307 phosphorylation levels (−163% vs. Ts-V, *p* = 0.02) in Ts-P mice (Figure 1F,G). These data suggest that brain insulin resistance develops in Ts2Cje mice brain while the KYCCSRK peptide administration rescues IR/IRS1 activation.

To test whether the observed KYCCSRK-induced IR and IRS1 activation was associated with the stimulation of the entire signaling, we evaluated AKT levels and activation since AKT is one of the main intracellular proteins activated in response to insulin [48,49]. Our results show no changes for AKT protein levels (Figure 1H), while increased AKT phosphorylation (evaluated as pAktS473/Akt ratio), and thus activation, in Ts-P mice, was observed (+319% vs. Ts-V, *p* = 0.05) (Figure 1I).

Finally, to better clarify the molecular mechanisms responsible for the observed effects mediated by the KYCCSRK administration in Ts2Cje mice, we focused on two key proteins regulating insulin signaling activation: the BVR-A and the phosphatidylinositol-3,4,5-trisphosphate 3-phosphatase (PTEN).

BVR-A is one of the main regulators of insulin signaling, that forms a regulatory loop together with IR and IRS1 [27,50], while downstream from IR/IRS1 it favors the activation of several proteins including ERK1/2, Akt, GSK-3β, PKCζ and mTOR [42,51,52,53,54]. As explained above, the KYCCSRK peptide corresponds to the C-terminal 7 residues of human BVR-A, so we were interested in understanding whether the peptide might affect mouse BVRA protein levels. We found no changes for BVRA either in Ts-V mice vs. Eu or in Ts-P mice (Figure 1J), suggesting that BVRA functions are preserved following the treatment.

PTEN is a phosphatase, that regulates the activation of the insulin signaling pathway by reducing levels of PI3K-derived phosphatidylinositol (3,4,5)-trisphosphate (PIP3), which promotes AKT activation [55,56]. Phosphorylation at the level of S380/T382/383, the sites evaluated in the present study, is responsible for PTEN inhibition [56]. No changes were observed for PTEN levels and activation (Figure 1K,L).

Together, these results highlight that KYCCSRK peptide administration rescues brain insulin signaling activation both at levels of IR/IRS1 and AKT, without affecting regulatory proteins, in agreement with previous observations in vitro [27,40] and in vivo [41]. Rescuing brain insulin signaling activation might promote neuroprotective effects by reducing AD neuropathological hallmarks in the brain, by improving synaptic plasticity mechanisms, and by stimulating cell energy metabolism, which are all processes known to be impaired in DS [4,57].

### 3.2. Intranasal KYCCSRK Administration Reduces the Accumulation of AD Neuropathological Hallmarks in Ts2Cje Mice

AD neuropathological hallmarks, such as APP, APP cleavage products, and TAU phosphorylation, accumulate quite early in the brain of human and mouse models of DS, representing neurotoxic stimuli [1,22,23,58]. Indeed, the APP gene is triplicated in DS and is a major risk factor for AD development in DS [57]. In addition, alterations of brain insulin signaling were proposed to accelerate the development of AD pathology by promoting either the aberrant cleavage of APP or TAU hyper-phosphorylation in the brain [10,59]. Hence, to understand whether KYCCSRK peptide administration in Ts2Cje mice could impact the accumulation of AD neuropathological hallmarks in the brain, APP full length (APP), APP cleavage products (APP-C83 and APP-C99), and TAU levels and phosphorylation were evaluated.

**Figure 2:**
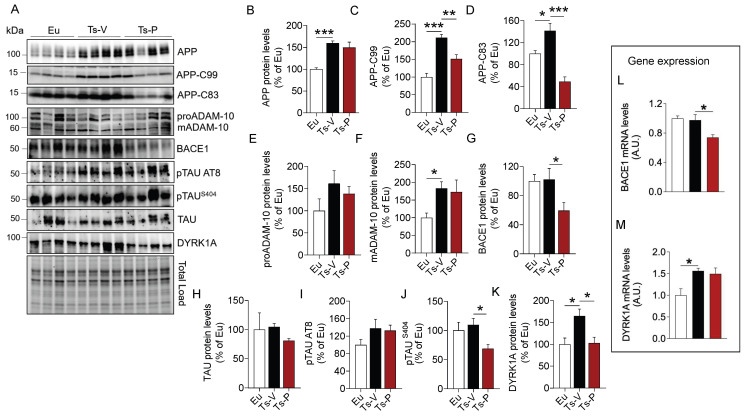
Reduced AD neuropathological hallmarks following KYCCSRK intranasal administration in Ts2Cje mice. APP, APP-CTFs, BACE1, TAU and DYRK1A protein levels and phosphorylation were evaluated in the frontal cortex of Eu and Ts2Cje (Ts-V) mice treated with vehicle (saline) and Ts2Cje (Ts-P) treated with KYCCSRK peptide (0.5 mM) for two weeks (n = 4/group). (**A**) Representative Western blot and total load images and densitometric evaluation of (**B**–**D**) APP, -C83 and -C99 fragments protein levels, (**E**,**F**) immature (proADAM-10) and mature (mADAM-10) forms of ADAM-10 protein levels, (**G**) BACE1 protein levels, (**H**–**J**) TAU protein levels, AT8 and S404 phosphorylation, and (**K**) DYRK1A protein levels. All densitometric values were normalized per total protein load and are given as a percentage of Eu set as 100%. In (**L**,**M**) BACE1 and DYRK1A mRNA levels are shown. Data are shown as mean ± SEM. One-way ANOVA with Dunnett test: * *p* < 0.05, ** *p* < 0.01, *** *p* < 0.001.

Our results confirmed that APP (+60% vs. Eu, *p* = 0.0009) (Figure 2B) and its cleavage products, i.e., APP-C99 (+111% vs. Eu, *p* = 0.0001) and APP-C83 (+42% vs. Eu, *p* = 0.02) are elevated in the brain of Ts-V mice (Figure 2C,D). Following the treatment, a significant reduction of both APP-C99 (−60% vs. Ts-V, *p* = 0.005) and APP-C83 (−92% vs. Ts-V, *p* = 0.0002) levels in Ts-P mice was observed (Figure 2C,D). To explain the mechanism through which the KYCCSRK peptide administration reduces APP processing, we evaluated protein levels of the enzymes α-secretase (ADAM-10) and β-secretase 1 (BACE1), that are responsible for the production of APP-C83 and APP-C99, respectively [60]. We observed a significant increase of the mature form (active) of ADAM-10 (mADAM-10) in Ts-V mice (+84% vs. Eu, *p* = 0.05), regardless of the immature form (proADAM-10), whose protein levels do not change among the groups (Figure 2E,F). No significant changes for active ADAM-10 were detected after the KYCCSRK peptide administration (Figure 2F). Conversely, a significant decrease of BACE1 protein levels in Ts-P (−43% vs. Ts-V, *p* = 0.013) was observed (Figure 2G).

We found no differences for TAU protein levels (Figure 2H), nor for TAU S202/T205 (AT8) phosphorylation (Figure 2I). Notably, a significant reduction of TAU S404 phosphorylation in Ts-P mice was observed (−40% vs. Ts-V, *p* = 0.04) (Figure 2J). To clarify this aspect, we evaluated DYRK1A protein, a kinase involved in TAU phosphorylation [61,62] whose gene is triplicated in DS [63,64]. Intriguingly, our data show that DYRK1A protein levels were elevated in Ts-V mice (+65% vs. Eu, *p* = 0.024), while a significant reduction was observed following the KYCCSRK administration (−61% vs. Ts-V, *p* = 0.025) (Figure 2K).

To better understand the process responsible for reduced BACE1 and DYRK1A protein levels following the treatment, their gene expression was evaluated. Reduced BACE1 mRNA levels in Ts-P (−23% vs. Ts-V, *p* = 0.01) were observed (Figure 2L). No differences were found for DYRK1A (Figure 2M).

In addition, we evaluated changes of proteins normally regulating autophagy. Autophagy is known to be impaired in the DS brain contributing to the accumulation of damaged proteins which form toxic aggregates triggering AD development in DS [65]. No significant changes were observed in Ts-P mice, possibly suggesting that KYCCSRK treatment does not affect the autophagic process, and thus the observed decrease of either APP-CTFs or pTAU levels might be mainly due to reduced BACE1 and DYRK1A protein levels (Supplementary Figure S3).

Finally, because the robust effects mediated by the KYCCSRK administration on APP/BACE1 and DYRK1A were quite intriguing and unexpected for us, we strengthened these observations by performing in vitro analyses. APP, APP-CFTs, BACE1, and DYRK1A levels were evaluated in Ts2Cje primary cortical neurons treated with different doses of KYCCSRK. Results collected in neurons confirmed what we have observed in Ts2Cje mice, by showing no changes for APP but reduced APP-CTFs, BACE1, and DYRK1A protein levels (Supplementary Figure S2).

In summary, the intranasal administration of the KYCCSRK peptide promoted an improvement in terms of AD pathology by reducing the amyloidogenic cleavage of APP as well as TAU phosphorylation likely mediated by reducing the expression levels of BACE1 and DYRK1A proteins.

### 3.3. Intranasal KYCCSRK Administration Reduces Oxidative Stress Levels in Ts2Cje Mice

**Figure 3:**
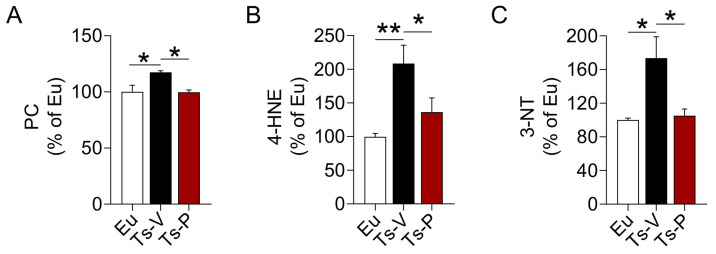
Reduced oxidative stress markers levels following KYCCSRK intranasal administration in Ts2Cje mice. (**A**) PC, (**B**) 4-HNE and (**C**) 3-NT levels were evaluated in the frontal cortex of Eu and Ts2Cje (Ts-V) mice treated with vehicle (saline) and Ts2Cje (Ts-P) mice treated with KYCCSRK peptide (0.5 mM) for two weeks (n = 4/group). Data were expressed as the percentage of Eu set as 100%. Data are shown as mean ± SEM. One-way ANOVA with Dunnett test: * *p* < 0.05, ** *p* < 0.01.

In previous works from our group, we highlighted a harmful synergistic effect between brain insulin resistance and oxidative stress in DS [22,23]. Both brain insulin resistance and increased oxidative stress levels in the brain were observed before the accumulation of APP-C99 and TAU phosphorylation, suggesting these events perhaps accelerate AD development in DS [22,23,66]. For that reason, we hypothesized that the beneficial effects of the KYCCSRK peptide treatment in Ts2Cje mice on brain insulin signaling might be associated with a reduction of oxidative stress levels. We evaluated the levels of three protein oxidation markers, named protein carbonyls (PC), 4-hydroxy-2-nonenal protein adducts (4-HNE), and protein-bound 3-nitrotyrosine (3-NT) in the frontal cortex of Ts2Cje and Eu mice. Our results show increased PC (+20%, *p* = 0.032) (Figure 3A), 4-HNE (+108%, *p* = 0.004) (Figure 3B) and 3-NT levels (+73%, *p* = 0.0152) (Figure 3C) in Ts-V mice that were significantly reduced following the KYCCSRK treatment in Ts-P mice: PC (−20%, *p* = 0.030), 4-HNE (−72%, *p* = 0.046) and 3-NT (−68%, *p* = 0.020) (Figure 3A–C).

### 3.4. Intranasal KYCCSRK Administration Increases Mitochondrial Complexes Levels in Ts2Cje Mice

Mitochondria are the powerhouse of cells responsible for the generation of ATP (through oxidative phosphorylation) and the maintenance of redox homeostasis, as well as for cell energy metabolism processes [67,68]. Genes involved in the oxidative phosphorylation process are mostly downregulated in DS consistent with the development of significant metabolic disturbances and increased oxidative stress levels in DS [69,70]. Furthermore, we found that reduced levels of mitochondrial complexes proteins were significantly associated with higher brain insulin resistance markers levels and increased oxidative stress levels both in human and mouse brains [22,23]. Hence, we hypothesized that the beneficial effects mediated by the KYCCSRK peptide on the insulin signaling pathway, AD neuropathological hallmarks, and oxidative stress levels in Ts2Cje mice, might be associated with an improvement of mitochondrial machinery.

**Figure 4:**
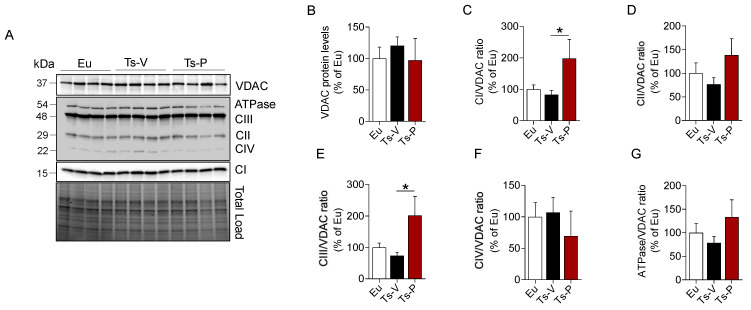
Changes of mitochondrial proteins following KYCCSRK intranasal administration in Ts2Cje mice. Mitochondrial OXPHOS complexes and VDAC protein levels were evaluated in the frontal cortex of Eu and Ts2Cje (Ts-V) mice treated with vehicle (saline) and Ts2Cje (Ts-P) mice treated with KYCCSRK peptide (0.5 mM) for two weeks (n = 4/group). (**A**) Representative Western blot and total load images and densitometric evaluation of (**B**) VDAC protein levels, (**C**–**G**) mitochondrial complexes (I–IV) and ATPase related to VDAC. All densitometric values were normalized per total protein load and are given as a percentage of Eu set as 100%. Data are shown as mean ± SEM. One-way ANOVA with Dunnett test: * *p* < 0.05.

We evaluated changes of mitochondrial complexes (I–IV), and ATP synthase (ATPase/CV) levels along with changes of voltage-dependent anion-selective channel (VDAC), a protein that plays a key role in maintaining high rates of oxidative phosphorylation [71]. We found that CI, CII, CIII, and ATPase levels showed a trend of reduction in the frontal cortex of Ts-V mice compared to Eu, although they did not reach statistical significance (Figure 4). Remarkably, KYCCSRK peptide administration promoted a significant increase of CI (+115% vs. Ts-V, *p* = 0.05) (Figure 4C) and CIII levels (+130%, *p* = 0.05) (Figure 4F) along with a trend observed for both CII and ATP synthase (+50% and +52%, respectively, vs. Ts-V) (Figure 4E–G). No changes for VDAC were observed.

Together, these results support the hypothesis that improving brain insulin signaling stimulates mitochondrial activation and cell energy metabolism in the Ts2Cje frontal cortex.

### 3.5. Intranasal KYCCSRK Administration Improves Synaptic Plasticity Mechanisms in Ts2Cje Mice

Brain insulin resistance impairs synaptic plasticity mechanisms, thus affecting learning and memory functions [10,72]. DS is characterized by a marked intellectual disability and studies in DS mouse models show that an imbalance between inhibitory vs. excitatory signals, likely due to an impairment of synaptic input integration [73], is responsible for cognitive decline in DS. Moreover, we previously demonstrated that brain insulin resistance is associated with alterations of mechanisms regulating synaptic plasticity both in DS human and mouse brains [20,22,23]. Hence, to determine whether the observed improvement of brain insulin signaling also was associated with an amelioration of synaptic plasticity mechanisms in Ts2Cje frontal cortex, changes of the GABA and AMPA currents along with synaptic proteins i.e., synaptophysin, PSD95, CamKIIα, and GluA1R, were evaluated.

**Figure 5:**
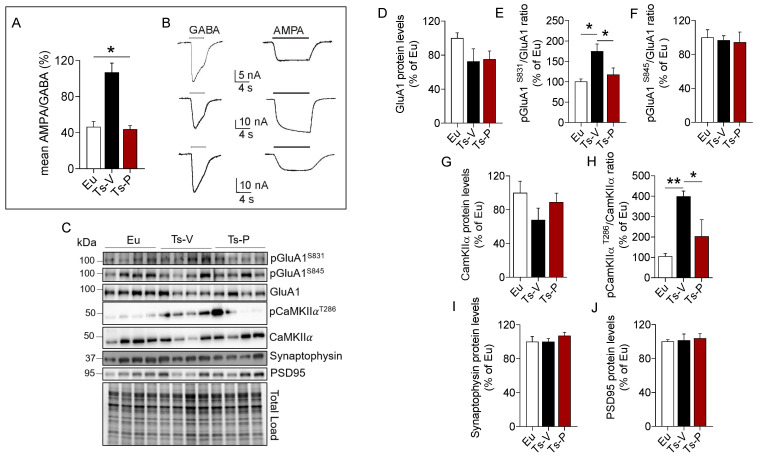
Improved synaptic plasticity mechanisms following KYCCSRK intranasal administration in Ts2Cje mice. AMPA and GABA currents in oocytes transplanted with cortical membranes along with proteins regulating synaptic plasticity mechanisms were evaluated in Eu and Ts2Cje (Ts-V) mice treated with vehicle (saline) and Ts2Cje (Ts-P) mice treated with KYCCSRK peptide (0.5 mM) for two weeks (n = 4/group). (**A**) Bars represent the mean ± SEM of the ratio between I_AMPA_ and I_GABA_ (reported as percentage) in Eu (oocytes n = 12), Ts-V (oocytes n = 20) and Ts-P (oocytes n = 13) mice, as shown. GABA 500 µM was applied for 4 s, while AMPA 20 μM plus CTZ were applied for 10 s. Holding potential was −60 mV and * shows statistical significance (Kruskal–Wallis One Way Analysis of Variance on Ranks with Dunn’s test, *p* < 0.05). The mean of I_AMPA_ was 7.2 ± 0.9 nA, 34.9 ± 3.9 nA, 11.4 ± 2.4 nA in Eu, Ts-V and Ts-P, respectively. The mean of I_GABA_ was 16.7 ± 2.3 nA, 35.3 ± 3.7 nA, 25.9 ± 4.8 in Eu, Ts-V and Ts-P, respectively. Note the similar values in Eu and Ts-P. (**B**) Representative current traces recorded in oocytes of the experiments shown in (**A**) as indicated; white bars, GABA evoked-currents; black bars, AMPA evoked-currents. (**C**) Representative Western blot and total load images and densitometric evaluation of (**D**) GluA1 total protein levels, (**E**,**F**) GluA1 phosphorylation at S831 and S845 (reported as pGluA1^S831^/GluA1 and pGluA1^S845^/GluA1 ratio); (**G**,**H**) CamKIIα protein levels and activation (evaluated as pCamKIIα^T286^/CamKIIα ratio); (**I**) synaptophysin and (**J**) PSD95 protein levels. All densitometric values were normalized per total protein load and are given as a percentage of Eu set as 100%. Data are shown as mean ± SEM. One-way ANOVA with Dunnett test: * *p* < 0.05 and ** *p* < 0.01.

We used the microtransplantation of exogenous cell cortical membranes in Xenopus oocytes that permits us to record evoked-currents from native receptors maintaining their original characteristics [45,46,47]. Using this simple but powerful approach, differences of the balance excitation/inhibition represented as AMPA/GABA currents percentage ratio were evaluated. A higher AMPA/GABA ratio in the Ts-V (106 ± 10.32%, oocytes n = 20) compared to the Eu mice (46.53 ± 5.84%, oocytes n = 12; *p* < 0.05) was observed. Notably, Ts-P mice showed a lower AMPA/GABA ratio compared to Ts-V (43.74 ± 4.1%, oocytes n = 13, *p* < 0.05), and were not statistically different from the Eu mice (Figure 5A), suggesting that KYCCSRK intranasal administration restores the physiological ratio between IAMPA e IGABA in Ts-P mice.

To explain changes of AMPA currents we looked at the AMPA receptor GluA1 subunit phosphorylation. Indeed, phosphorylation of the GluA1 subunit regulates activity-dependent AMPA receptor trafficking by the mean of two phosphorylation sites, i.e., S845 and S831 [74,75,76]. S845 phosphorylation promotes GluA1 surface expression and increases channel open-probability, while S831 phosphorylation augments the single-channel conductance [74,75,76]. Our data show no differences for GluA1 protein levels (Figure 5D). GluA1 S831 phosphorylation was significantly increased in Ts-V mice (+75% vs. Eu, *p* = 0.008), while it reduces to levels comparable to those observed in Eu mice after the KYCCSRK administration in Ts-P mice (−60% vs. Ts-V, *p* = 0.03) (Figure 5E). No significant changes for GluA1 S845 phosphorylation were observed (Figure 5F). CamKIIα is responsible for GluA1 S831 phosphorylation and changes in CamKIIα activity contribute to the turnover and modulation of GluA1 at the plasma membrane [77]. Our results show no changes for CamKIIα protein levels (Figure 5G), while a consistent CamKIIα activation (increased T236 phosphorylation) is observed in Ts-V mice (+294% vs. Eu, *p* = 0.004), that is significantly reduced following KYCCSRK treatment (−196% vs. Ts-V, *p* = 0.03) in Ts-P mice (Figure 5H). No changes for synaptophysin (pre-synaptic) and PSD95 (post-synaptic) were found (Figure 5I,J).

Overall, these data suggest that an imbalance between inhibitory vs. excitatory currents likely driven by an increased AMPA receptor conductance (triggered by CamKIIα-mediated GluA1 S831 phosphorylation) can be observed in Ts2Cje mice frontal cortex, while the KYCCRSK intranasal administration efficiently restores such alterations.

### 3.6. Correlation Analyses and PCA

**Figure 6:**
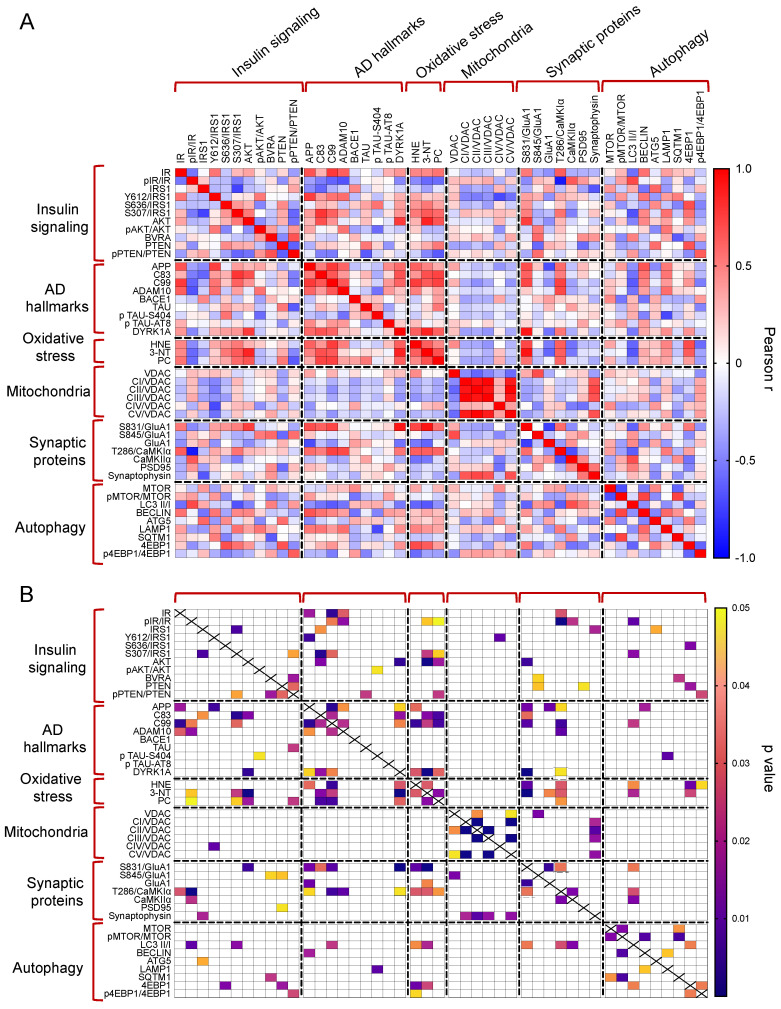
Correlation analyses. Pearson correlation analyses were performed to explore associations among all the proteins measured in in our work. Correlations are shown as correlation matrix. In (**A**) Pearson r values are reported. In (**B**) significant correlations are indicated.

**Figure 7:**
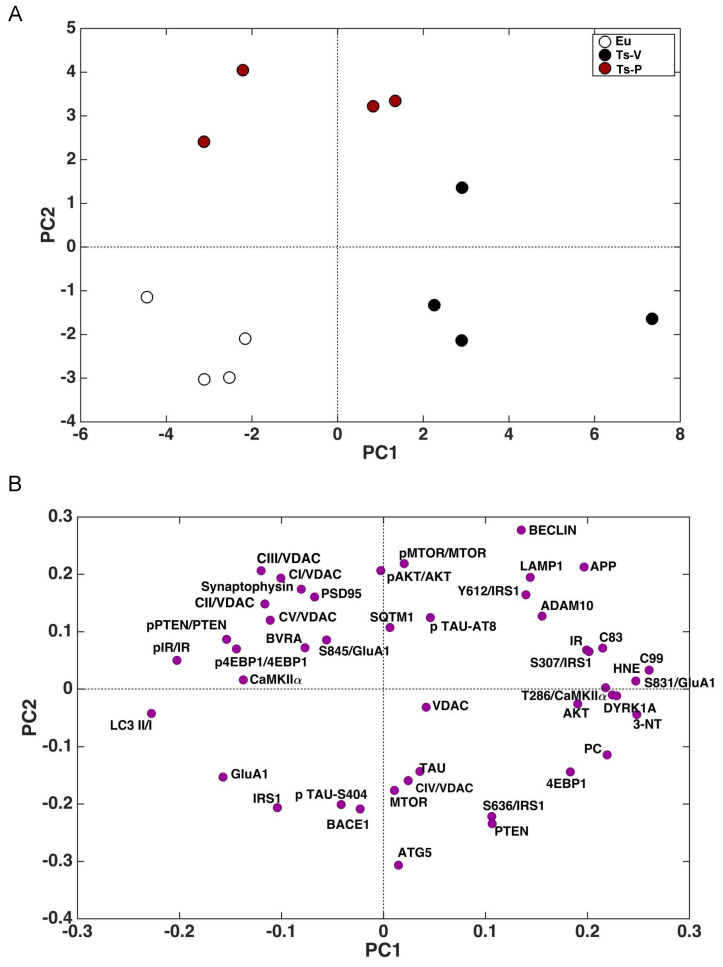
Principal component analysis results. Principal component analysis (PCA) was performed on the results collected in mice to determine variance contribution of the components associated with the observed neuroprotective effects promoted by KYCCSRK administration in Ts-P mice. Score plots (**A**) and loadings (**B**) graphs are shown.

The whole set of measured proteins was used to build a correlation matrix to look at any significant association. Results are reported in Figure 6A,B. Among significant correlations, we observed that IR activation is negatively associated with 3-NT (r = −0.58, *p* = 0.04); and CamKIIα activation (T236/CamKIIα, r = −0.90, *p* < 0.001) levels while IRS1 inhibition (S307/IRS1) is positively associated with 3-NT (r = 0.64, *p* = 0.02). Furthermore, among the proteins involved in AD neuropathology, APP-C99 levels are positively associated with CamKIIα activation (r = 0.63, *p* = 0.03) while DYRK1A protein levels are positively associated with oxidative stress markers (HNE: r = 0.63, *p* = 0.02; 3-NT: r = 0.87, *p* < 0.001; PC: r = 0.64, *p* = 0.03), GluA1 S831 phosphorylation (S381/GluA1, r = 0.91, *p* < 0.001) and CamKIIα activation (r = −0.54, *p* = 0.04). Morevoer, CamKIIα activation is positively associated with GluA1 S831 phosphorylation (r = 0.57, *p* = 0.003). Then, the multivariate data were arranged in a matrix which was then subjected to a principal component analysis (PCA). Resulting scores plot is displayed in Figure 7. PCA outcomes show how the first principal component (PC1) differentiates between Ts-V (at positive scores) and Eu and Ts-P mice (at negative scores), these latter sharing similar features. In particular, by looking at the loadings (Figure 7A), it appears that Ts-V mice differ from Eu and Ts-P mice because they are characterized by brain insulin resistance (lower pIR/IR and higher pIRS1S307/IRS1, and pIRS1S636/IRS1), the accumulation of AD-associated neuropathological hallmarks (higher APP, APP-C83, APP-C99, DYRK1A, and TAU), increased oxidative stress levels (higher PC, HNE and 3-NT) and the aberrant activation of proteins regulating synaptic plasticity mechanisms (higher pGluA1 S831, pCaMKIIα T286). Moreover, it is possible to differentiate Eu from Ts-P mice along the second principal component (PC2), since the former falls at negative scores and the latter at positive scores. In this case, it is also possible to interpret the observed differences in terms of the measured variables, by inspecting the loadings plot in Figure 7B. In particular, Ts-P mice are characterized by an improvement of brain insulin signaling activation (higher pAKT/AKT), an amelioration of mitochondrial bioenergetics (higher CV/VDAC, CIII/VDAC, CII/VDAC, and CI/VDAC) and an improvement of synaptic plasticity mechanisms (higher PSD95 and Synaptophysin).

## 4. Discussion

Growing evidence suggests that brain insulin signaling, other than having a role in the regulation of cerebral metabolism, regulates key molecular pathways involved in mood, behavior, and cognition [48], that are impaired following development of brain insulin resistance. In general, insulin resistance is defined as the reduced response to insulin by target cells, due to reduced IR levels and/or activation or increased IRS1 inhibitory phosphorylation that finally results in impaired glucose uptake and cell metabolism [49,50,51]. Moreover, brain insulin resistance mediates synaptotoxic effects in several ways leading to (1) the accumulation of AD neuropathological hallmarks [10,52,53,54]; and (2) synapse loss, impaired autophagy, and increased neuronal apoptosis [10,55,56]. These observations collected in vitro and in animal models have been strengthened by clinical studies reporting that the failure in brain energy metabolism responsible for the cognitive decline during aging or AD could be driven by the development of brain insulin resistance particularly at the early stages [10,57].

We previously reported on the accumulation of markers of brain insulin resistance, such as reduced IR protein levels and increased IRS1 inhibition, in the brain of young DS individuals before AD development [23]. Furthermore, we proposed that the development of brain insulin resistance triggers AD onset in DS since reduced IR protein levels along with the impairment of insulin signaling are associated with a great amyloidogenic cleavage of APP (increased APP C99 levels) in DS < 40 years old but not in age-matched controls [23].

Here we show for the first time that brain insulin signaling activation can be rescued by means of the intranasal administration of the KYCCSRK peptide in Ts2Cje mice. Our results demonstrate that KYCCSRK leads to IR activation along with increased activation of IRS1 and downstream targets, i.e., AKT in the frontal cortex. The activation of brain insulin signaling can be observed also in Eu mice, suggesting that the peptide is able to exert its effects under physiological conditions. These results agree with the role proposed for KYCCSRK promoting insulin-independent activation of IR and AKT in several cell lines and to an extent like results observed with insulin stimulation [27,40] as well as in vivo [41]. Furthermore, the observed increased activation of IR and AKT following KYCCSRK administration in Ts2Cje mice strengthens the idea that such a peptide can cross the BBB and reach the frontal cortex when administered through the intranasal route.

Defects of insulin signaling have been shown to co-occur with Aβ plaques and Tau phosphorylation in the temporal lobe, hippocampus, and cerebellum [14,18,52,58]. Brain insulin resistance triggers the accumulation of APP toxic fragments (e.g., Aβ and -C99) either by favoring an aberrant APP cleavage or by inhibiting their clearance [14,15,16,53,59,60]. Increased APP-C83 and -C99 levels in human DS brains were observed to an even greater extent than those found in AD brains [61]. Moreover, the accumulation of both APP-C83 and APP-C99 was shown to contribute to endo-lysosomal abnormalities and the buildup of other oxidized substrates that are responsible for damaging cellular components in the DS brain [62,63,64]. Results of the current study show that KYCCSRK peptide, by rescuing insulin signaling activation, decreases the levels of APP-CTFs fragments (both -C83 and -C99) within the frontal cortex regardless of APP. APP-C83 levels are reduced independently of ADAM10 protein levels, and possibly by increased C83 clearance following the KYCCSRK treatment. The improvement of insulin signaling seems to have a role, in agreement with previous studies showing that insulin administration reduces APP CTFs levels both in vitro and in vivo [65,66]. Indeed, a strong positive correlation between C83 and IRS1 inhibition (S307) was found in our study. We observed that the levels of BACE1 protein—the secretase responsible for the generation of APP-C99 fragment in the amyloidogenic pathway [67]—are consistently decreased in the Ts2Cje mice following KYCCSRK administration, which may explain reduced APP-C99 levels observed in Ts-P mice. Furthermore, in a recent study, BACE1 was shown to impair IR activation [68]. Hence, it might be hypothesized that reduced BACE1 protein levels also contribute to the observed activation of IR in the frontal cortex following KYCCSRK administration in Ts-P mice. Lower BACE1 protein levels seem to result from reduced transcription (reduced mRNA levels). Whether the peptide on its own or the activation of insulin signaling represses *BACE1* gene transcription was not established in this paper, and is out of the scope of this study. This is an intriguing aspect that deserves further investigations and studies on the topic are ongoing in our laboratory.

Other than APP-associated neuropathology, KYCCSRK treatment reduced TAU phosphorylation in the frontal cortex of Ts-P mice. This result is remarkable because it underlies protective mechanisms of the KYCCSRK peptide in terms of TAU biology in DS. Lack of differences between Eu and Ts-V mice in our study is not surprising, considering that changes of TAU phosphorylation are both age- and brain region-specific due to the genetics of the different DS mouse models used in the literature [69,70,71]. To better understand the mechanisms responsible for reduced TAU phosphorylation, we looked at the DYRK1A protein. DYRK1A is abundantly expressed in the brain and interacts with numerous cytoskeletal, synaptic, and nuclear proteins in neurons, including TAU [72]. Interestingly, the *DYRK1A* gene is triplicated in DS [73,74], having a role in the observed impairment in neuronal development and neuronal activities [73,74]. Moreover, DYRK1A protein through its kinase activity promotes TAU phosphorylation in DS [73,75]. KYCCSRK led to reduced DYRK1A protein levels in the frontal cortex of Ts-P mice. Notably, overexpression of DYRK1A in peripheral organs was related to diabetes phenotypes [76], while pharmacological inhibition of DYRK1A leads to an improved glycemic control in both mice and human cells [77,78,79]. Together, the current results and previous observations further support the hypothesis that elevated DYRK1A protein levels might have a role in brain insulin resistance development and AD pathological hallmarks accumulation in DS.

KYCCSRK neuroprotective effects include reduced oxidative stress levels observed in the frontal cortex of Ts-P mice. Previous studies demonstrated that oxidative stress is an early event in DS [22,60,80,81], likely representing a key factor in the development of a variety of pathological phenotypes. Furthermore, the overexpression of some HSA21 genes, e.g., *APP* and *DYRK1A,* and the dysregulation of gene/protein expression associated with the trisomy contribute to the increase of oxidative stress in DS [82,83,84], suggesting that reduced APP amyloidogenic cleavage and DYRK1A protein levels might account for the observed decrease of oxidative stress levels in Ts-P frontal cortex. The negative correlations found between DYRK1A, and oxidative stress markers reinforce this hypothesis.

The antioxidant properties of KYCCSRK also rely on the improved activation of insulin signaling. Insulin signaling plays a pivotal role in the maintenance of mitochondrial bioenergetics [85,86,87]. Conversely, the development of brain insulin resistance would promote mitochondrial dysfunctions responsible for reduced energy production, in turn, associated with an increase of reactive oxygen/nitrogen species formation, finally leading to the oxidative/nitrosative damage of mitochondria as well as other cellular components [86]. Brain insulin resistance and increased oxidative stress markers levels are tightly associated with cognitive dysfunctions [88,89,90,91,92,93] and also in DS [22,23,81], suggesting a close link exists among these events. Intriguingly, insulin deprivation led to increased oxidative stress levels in the mouse brain [94], while intranasal insulin treatment improved mitochondrial functions and reduced oxidative stress levels both in healthy mouse brains [94] and in an AD mouse model [27]. In agreement with these findings, increased levels of mitochondrial complexes were observed in Ts-P mice frontal cortex samples following KYCCSRK administration, strengthening the role for improved insulin signaling activation in cell metabolism and energy production [8]. Indeed, our results highlight a robust effect for CI and CIII levels, which are two complexes found significantly reduced in the DS brain, playing a central role in mitochondrial dysfunction, energy production, and oxidative stress [82,95].

We underline that the neuroprotective effects elicited by the intranasal administration of the KYCCSRK peptide positively impact on brain plasticity, which is known to be finely modulated by insulin [96]. In fact, insulin regulates synaptic plasticity mechanisms by upregulating synaptic receptor subunits and SNARE proteins responsible for neurotransmitter release [48]. Conversely, alterations of insulin signaling in the central nervous system impair brain plasticity, promote synapse loss and neurodegeneration, and accelerate brain aging [97]. Notably, GABAergic transmission is altered in DS, and several studies suggest an excessive activity of inhibitory circuits in this condition [98]. In particular, DS mouse models show a plethora of alterations—including increased seizures incidence, sleep alterations, and hyperactivity in locomotor behavior—that underlie an imbalance between inhibitory vs. excitatory signals, likely due to an impairment of synaptic inputs integration [99]. Our results agree with this hypothesis since an impairment of the I_AMPA_/I_GABA_ currents ratio in favor of excitatory currents (I_AMPA_) was observed in Ts-V mice, while the KYCCSRK administration restored such alteration in Ts-P mice. Regulation of AMPAR functions is highly dynamic in many different forms of synaptic plasticity, including long-term potentiation and depression (LTP/LTD) and homeostatic synaptic plasticity [100,101]. Mechanistically, restoration of the I_AMPA_/I_GABA_ currents ratio seems to be due to the reduced GluA1 S381 phosphorylation, which decreases GluA1 conductance thus reducing AMPA currents [100,101] in Ts-P mice. GluA1 phosphorylation is regulated by insulin signaling (reviewed in [102]). Notwithstanding, studies evaluating the effects of exogenous insulin administration on AMPAR-mediated glutamatergic transmission raised discordant conclusions showing either increased or reduced GluA1 phosphorylation (reviewed in [102]). We found strong positive correlations among DYRK1A, pCamKIIα and pGluA1 S381 suggesting that reduced DYRK1A levels in Ts-P mice might be responsible for reduced pCamKIIα-mediated GluA1 S381 phosphorylation. This is an intriguing hypothesis because DYRK1A plays a pivotal role in synaptic plasticity mechanisms [73,75] and particularly at the glutamatergic synapses [103]. In previous works from Herault’s group, it has been shown that DYRK1A interacts with synaptic proteins, among which CamKIIα—a kinase for GluA1 S831 residue [104] —was identified [103,105]. Overexpression of DYRK1A was associated with increased CamKIIα protein levels in Dp1Yey mice (a murine model for DS), that returned to normal following reducing DYRK1A gene expression [103]. While the activation of CamKIIα was not evaluated in these studies, it is conceivable to think that higher CamKIIα levels parallel higher CamKIIα activation in Dp1Yey mice. We did not observe increased CamKIIα protein levels in the cortex of Ts-V mice, but an increased CamKIIα activation, that to our opinion is responsible for increased GluA1 S831 phosphorylation and impaired I_AMPA_/I_GABA_ currents ratio in Ts-V mice. This hypothesis is further reinforced by the observation that DYRK1A protein while interacting with CamKIIα does not interact with GluA1 in wild-type mice brain extract [103]. Hence, overexpression of DYRK1A recruits synaptic proteins, e.g., CamKIIα, impairing their activity in the DS brain, while normalizing DYRK1A gene expression and protein levels is associated with neuroprotective effects [103,105,106].

Finally, we acknowledge that one limitation of our study is the relatively small group of animals used to test the neuroprotective effects of the KYCCSRK peptide. However, despite the sample size, consistent effects on outcome measures suggest this may be a robust effect.

## 5. Conclusions

**Figure 8:**
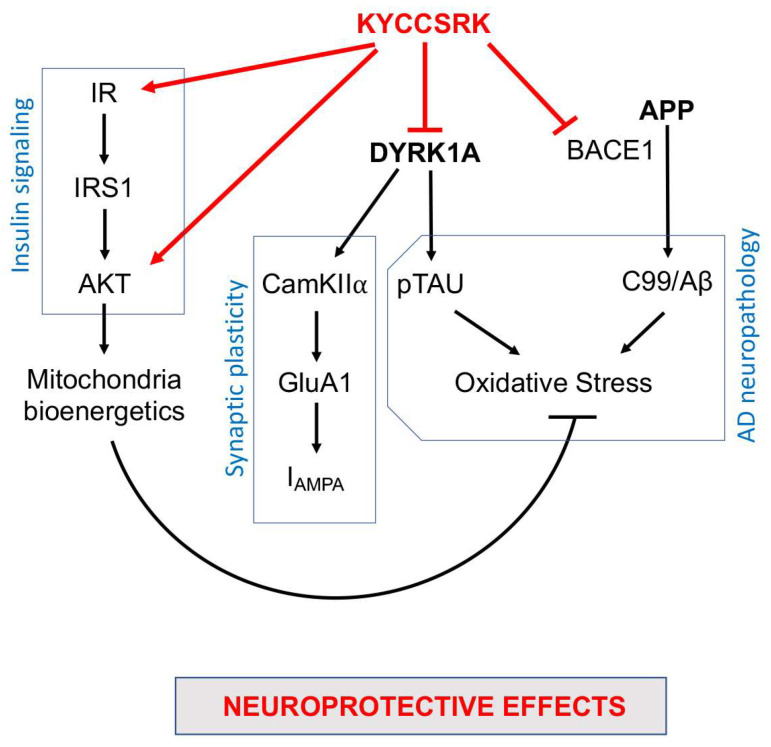
Schematic representation of the molecular mechanisms associated with the neuroprotective effects mediated by the intranasal administration of the KYCCSRK peptide in Ts-P mice. Arrows: stimulation; lines: inhibition.

In conclusion, our work identified novel neuroprotective properties for the KYCCSRK peptide that other than rescuing brain insulin signaling activation in Ts-P mice also promotes an amelioration of some pathways involved in AD neuropathology development (Figure 8). Moreover, the results of the current study further strengthen the idea that the dysfunction of the insulin signalling pathway crosses with dysfunctions of proteins encoded by genes on HSA21, e.g., *DYRK1A* and *APP*, thus contributing to worsening a pre-existing condition defined on a genetic background in the DS brain. Hence, KYCCSRK might represent a new therapeutic opportunity to restore the DS brain functions affected by the above-mentioned alterations. This aspect is fascinating especially in light of the role of the insulin signaling pathway in regulating energy metabolism and cognitive functions and of the fact that accumulation of brain insulin resistance markers is evident in children and adolescents with DS [20], and thus several years before AD development. Finally, the significant effects obtained relative to DYRK1A and BACE1/C99 represent promising observations in searching for new molecules for ameliorating intellectual disability and fighting AD development in DS.

## Figures and Tables

**Table 1:** Antibodies used in the current work.

Name	Code	Company	Dilution
3-NT	SAB5200009	Sigma-Aldrich	1:1000
4-HNE	NB10063093	Novus	1:2000
4EBP1 (P1)	sc-9977	SANTA CRUZ	1:1000
Akt	AM-1011	ECM	1:1000
ADAM10	Ab227172	Abcam	1:1500
APP	A8717	Sigma-Aldrich	1:10,000
APP-C99	MABN380	Sigma-Aldrich	1:1000
ATG5	sc-133158	SANTA CRUZ	1:1000
BACE1	sc-33711	SANTA CRUZ	1:1000
Beclin	3738	Cell signaling	1:1000
BVR-A	Ab90491	Abcam	1:5000
CamKIIα	Sc-32288	Santa Cruz	1:1000
DYRK1A	8765S	Cell signaling	1:1000
GluA1	2263	Millipore	1:1000
GLUT 4 (IF-8)	sc 53566	SANTA CRUZ	1:1000
IR	3020S	Cell signaling	1:1000
LC3B	NB-1002220	Novus	1:1000
NdufB6	PA5-60579	ThermoFisher	1:2000
OXPHOS	Ab110411	ABCAM	1:2000
p4EBP1 (T36)	sc-18080-R	SANTA CRUZ	1:500
pAkt (S473)	44621	Invitrogen	1:1000
pAS160 (T642)	GTX 55118	Gene Tex	1:1000
pCamKIIα (T286)	12716	Cell signaling	1:1000
pGluA1 (S831)	4-833	Millipore	1:1000
pGluA1 (S845)	8084	Cell signaling	1:1000
pIR (Y1146/Y1150/Y1151)	GTX25681	Gene Tex	1:1000
pIRS1 (S307)	2381	Cell signaling	1:1000
pIRS1 (S636)	GTX 32400	Gene Tex	1:1000
pIRS1 (Y612)	GTX24868	Gene Tex	1:1000
pmTOR (S2448)	5536S	Cell signaling	1:1000
pPTEN (S380/T382/383)	sc-101789	SANTA CRUZ	1:500
Protein Carbonyl	S7150	Sigma-Aldrich	1:5000
PSD95	D27E11	Cell signaling	1:1000
pTAU (AT8)	MN1020	Invitrogen	1:2000
pTAU (S404)	Ab92676	ABCAM	1:1000
PTEN (A2B1)	sc-7974	SANTA CRUZ	1:1000
SQSTM1	GTX100685	Genetex	1:1000
Synaptophysin	8049	Abcam	1:1000
Tau	orb-46243	Biorbyt	1:1000
VDAC	PA1-954A	Invitrogen	1:1000

## Data Availability

Data are those available within the article and the Supplementary Materials.

## Supplementary Materials

The following supporting information can be downloaded at: https://www.mdpi.com/article/10.3390/antiox12010111/s1, Figure S1: Activation of brain insulin signaling in response to increasing doses of KYCCSRK peptide administered through the intranasal route in Eu mice; Figure S2: Reduced AD neuropathological hallmarks following KYCCSRK treatment in primary cortical neurons isolated from Ts2Cje mice; Figure S3: The KYCCSRK peptide intranasal administration in Ts2Cje mice does not promote changes of proteins regulating autophagy.
