# Supplementary material for: Intranasal Administration of KYCCSRK Peptide Rescues Brain Insulin Signaling Activation and Reduces Alzheimer’s Disease-like Neuropathology in a Mouse Model for Down Syndrome
Source: Antioxidants (Basel). 2023 Jan 2;12(1):111. doi: 10.3390/antiox12010111 (PMC9854894; doi:10.3390/antiox12010111)
Supplement: Supplementary file 1 [file antioxidants-12-00111-s001.zip › antioxidants-2138842-supplementary.pdf]

antioxidants

MDPI

Supplementary Figures

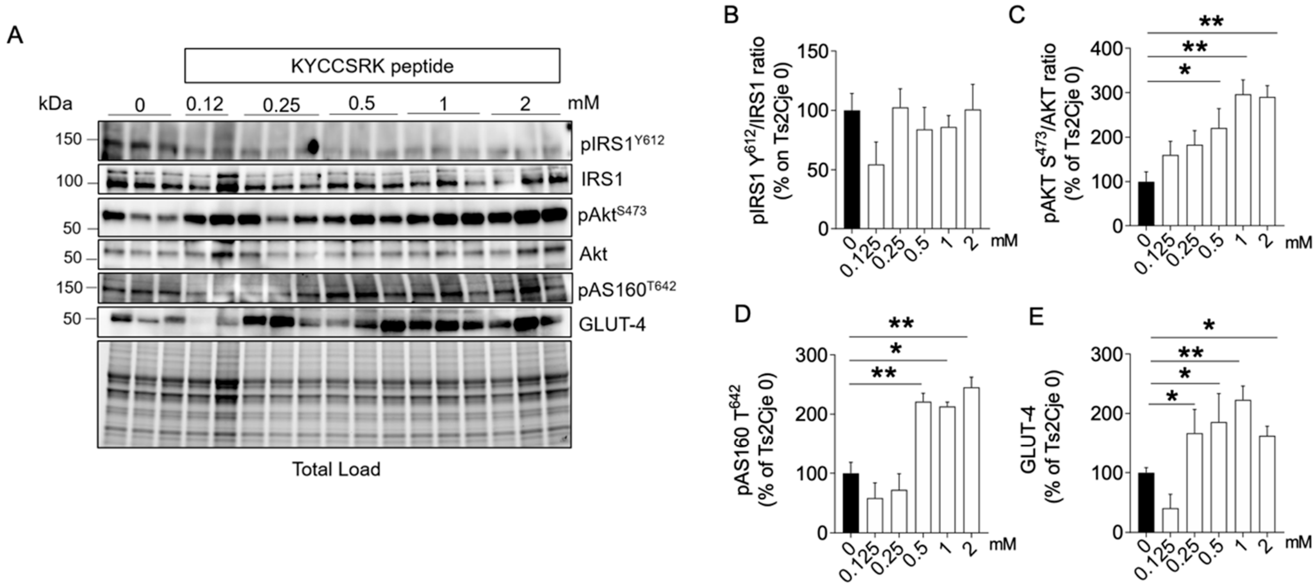

Supplementary Figure S1. Activation of brain insulin signaling in response to increasing doses of KYCCSRK peptide administered through the intranasal route in Eu mice. The KYCCSRK peptide dose used for the treatment described in the main text was chosen from a dose-response pilot study. Eu mice were treated with an intranasal administration of vehicle (saline) or different doses (0.12, 0.25, 0.5, 1 and 2 mM) of KYCCSRK peptide and sacrificed after 1 hour (n = 3/group). Our data show that 0.5 mM KYCCSRK peptide was able to induce an increased AktS437 phosphorylation along with an increase of Akt substrate 160 (AS160) T642 phosphorylation, which is responsible for translocation of GLUT-4-containing vesicles to the plasma membrane and increased insulin-dependent glucose transporter-4 (GLUT-4) protein levels. Together these observations suggest that the KYCCSRK peptide promotes the activation of brain insulin signaling when administered through the intranasal route. (A) Representative Western blot and total load images and densitometric evaluation of (B) IRS1 activation (pIRS1Y612/IRS1), (C) AKT activation (pAKTS473/AKT), (D) AS160 activation (pAS160T642) and (E) GLUT-4 levels. Protein levels were normalized per total protein load. IRS1 and AKT-associated phosphorylations were normalized by considering the respective protein levels and are expressed as the ratio between the phosphorylated form and the total protein levels. To assess AS160 protein levels, we tested commercially available antibodies, but none of them produced a satisfactory signal. All densitometric values were normalized per total protein load and are given as percentage of Eu set as 100%. Data are shown as mean ± SEM. One-way ANOVA with Dunnett test: *p <0.05, **p<0.01, ***p<0.001.

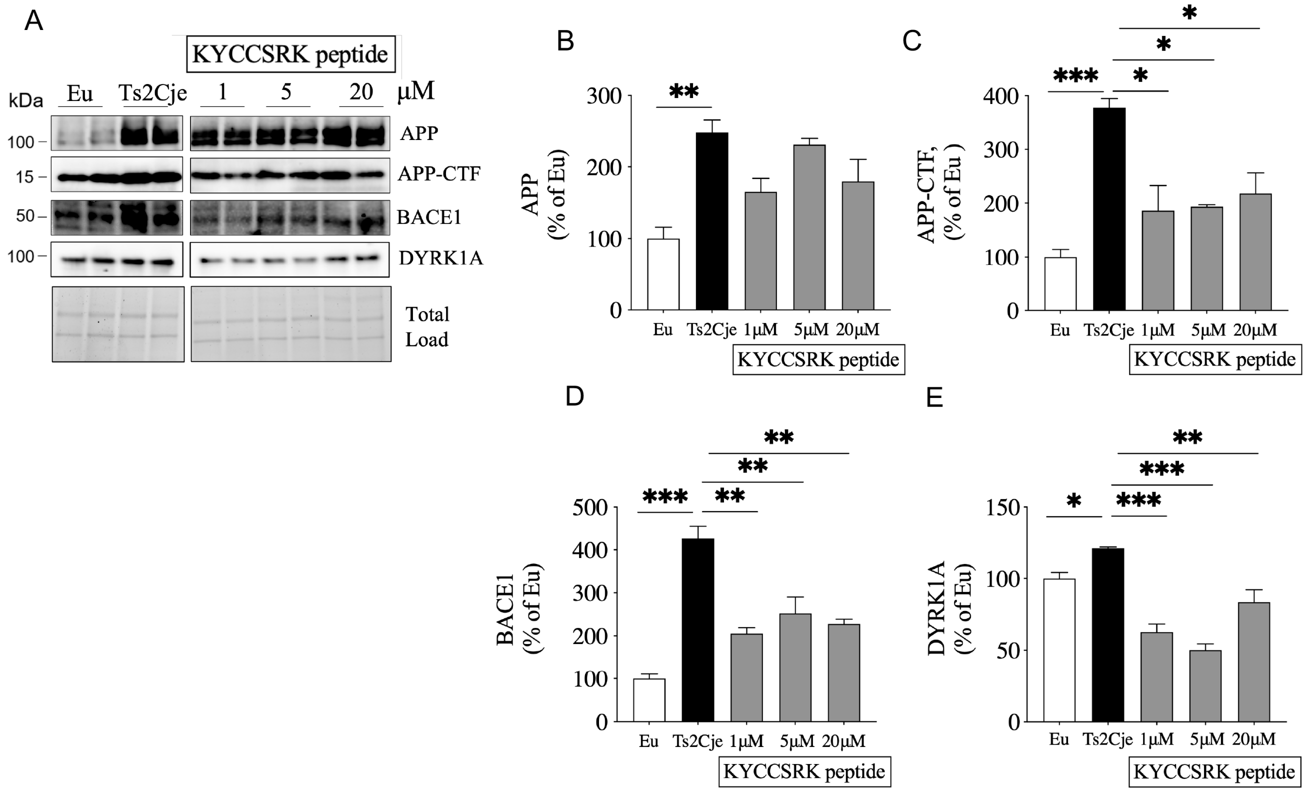

Supplementary Figure S2. Reduced AD neuropathological hallmarks following KYCCSRK treatment in primary cortical neurons isolated from Ts2Cje mice. To strengthen the results obtained by the intranasal treatment with KYCCSRK peptide on AD neuropathological hallmarks in Ts2Cje mice, we treated cortical neurons isolated from Ts2Cje mice with three different doses (1, 5 and 20 μM) of peptide for 24 hours. Our data confirmed the significant increase in APP (+147.9% vs Eu, p=0.052), APP CTFs (+278 %, p=0.0009), BACE1 (+326% vs Eu, p=0.0001) and DYRK1A (+21% vs Eu, p=0.02) levels in neurons isolated from Ts2Cje. Interestingly, when Ts2Cje neurons treated with the KYCCSRK peptide show a significant reduction of: (i) APP CTFs (1μM, -191% p=0.014; 5μM, -184% p=0.012; and 20μM, -154% p= 0.024 vs Ts2Cje), (ii) BACE1 protein levels (1μM, -221% p=0.0016; 5μM, -174% p=0.005; and 20μM, -198% p= 0.002 vs Ts2Cje) and (iii) DYRK1A protein levels (1μM, -58% p<0.001; 5μM, -71% p<0.0001; and 20μM, -38% p<0.01 vs Ts2Cje). (A) Representative Western blot and total load images and densitometric evaluation of (B) APP protein levels, (C) APP-CTFs, (D) BACE1 and (E) DYRK1A protein levels. All densitometric values were normalized per total protein load and are given as percentage of Eu primary neurons set as 100%. Data are shown as mean ± SEM (n= 3 independent cultures/group) One-way ANOVA with Dunnett test: *p <0.05, **p<0.01, ***p<0.001.

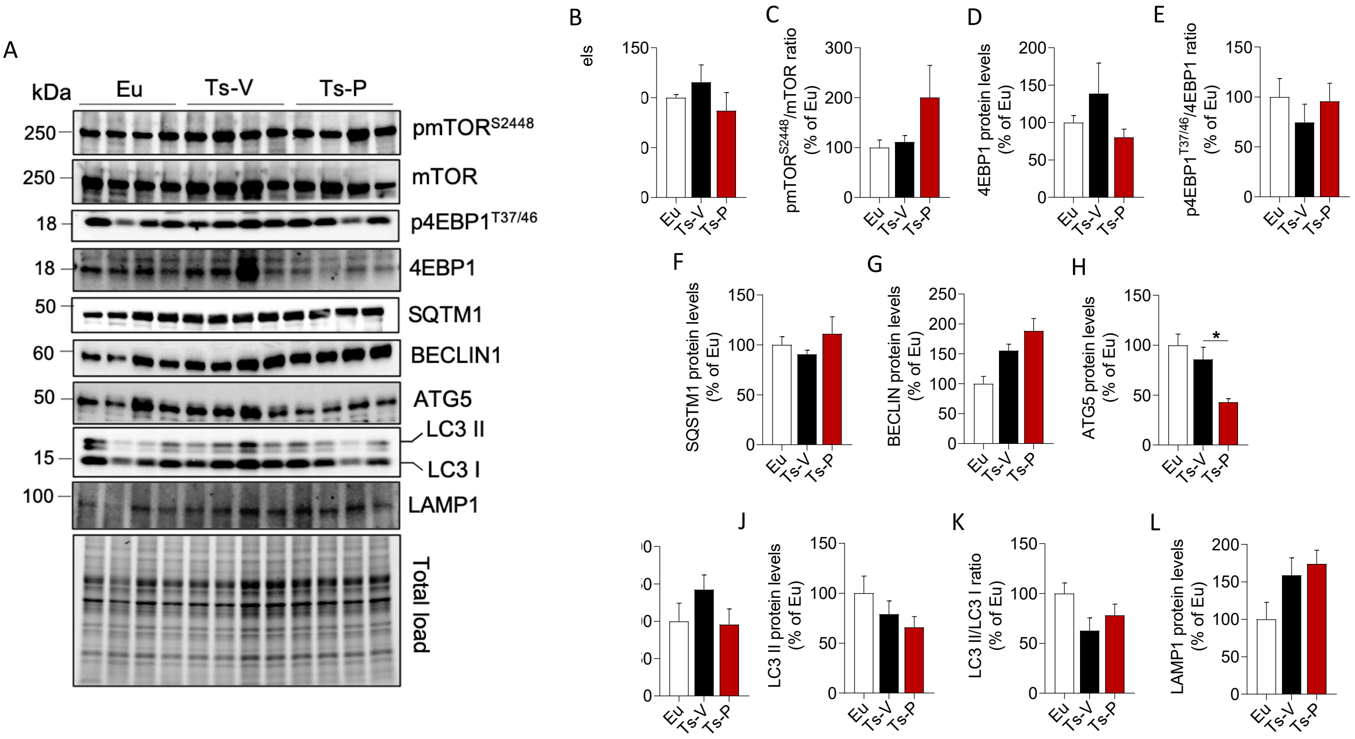

Supplementary Figure S3. The KYCCSRK peptide intranasal administration in Ts2Cje mice does not promote changes of proteins regulating autophagy. Autophagy is an important cellular process, whereby cell debris, including proteins and organelles, are degraded, and recycled to prevent the buildup of protein clusters and aggregates. The alteration of autophagy is reported in various neurodegenerative and lysosomal storage disorders and has been extensively demonstrated in DS. We measured: (a) proteins that were involved in the regulation of autophagy process, as Sequestosome 1 (SQSTM1), mTOR (mTORS2448/mTOR) and its downstream target activation (4EBP1T37/46/4EBP1) and (b) several markers that participate in the autophagosome formation, as BECLIN, microtubule-associated protein 1A/1B-light chain 3 (as ratio of LC3II/I), Lysosomal-associated membrane protein 1 (LAMP1) and Autophagy Related 5 (ATG5). Our results show no significant changes in the activation of mTOR pathway nor in Ts-V compared to Eu mice neither in Ts-P. No changes are reported also in SQSTM1 protein levels, as indicator of autophagic flux. Thus, the KYCCSRK peptide treatment didn't exert any effects on the autophagosome formation process in the brain of Ts-P mice. (A) Representative Western blot and total load images and densitometric evaluation of (B and C) mTOR protein levels and its activation (measured as ratio of mTORS2448/mTOR), (D and E) 4EBP1 protein levels and phosphorylation (measured as ratio of 4EBP1T37/46/4EBP1), (F, G and H) SQTM1, BECLIN and ATG5 protein levels, (I, J and K) LC3II/I ratio and (L) LAMP1 protein levels, markers of autophagy flux. All densitometric values were normalized per total protein load and are given as percentage of Eu set as 100%. Data are shown as mean ± SEM. One-way ANOVA with Dunnett test.
